# Early palliative care decision in patients with primary brain tumor reduces emergency department visits and hospitalization at the end of life

**DOI:** 10.1007/s11060-025-05377-3

**Published:** 2025-12-24

**Authors:** Nelli-Sofia Nåhls, Anu Anttonen, Pauliina Kitti, Riikka-Leena Leskelä, Outi Akrén, Tiina Saarto, Timo Carpén

**Affiliations:** 1 Department of Oncology, Vaasa Central Hospital, The Wellbeing Services County of Ostrobothnia, Vaasa, Finland; 2 Comprehensive Cancer Centre, Department of Oncology, University of Helsinki, Helsinki, Finland; 3 Comprehensive Cancer Center, Department of Radiotherapy, Helsinki University Hospital, Helsinki, Finland; 4 Nordic Healthcare Group, Helsinki, Finland; 5 Palliative Care Center, Comprehensive Cancer Center, Helsinki and Palliative Center, Helsinki University Hospital, University Hospital of Turku and Turku University, Turku, Finland; 6 Palliative Care Center, Comprehensive Cancer Center, Faculty of Medicine, Helsinki University Hospital, Helsinki University, Helsinki, Finland

**Keywords:** Palliative care decision, Emergency department, Hospitalization, End of life

## Abstract

**Purpose:**

Palliative care (PC) remains underutilized among patients with primary brain tumors, despite the life-threatening nature of the disease and the high symptom burden. This study aimed to assess how the timing of a PC decision (i.e., terminate life-prolonging anticancer treatments) is associated with emergency department visits and hospitalizations at the end of life (EOL).

**Methods:**

This single-center retrospective cohort study included adult patients (≥ 18 years) with primary brain tumor treated at the Comprehensive Cancer Center of Helsinki University Hospital during 2017–2018 who died by the end of 2018. Patients were categorized into “early PC decision” (> 30 days before death) or “late/no PC decision” (≤ 30 days or no decision). We extracted data on hospital resource use from electronic medical records.

**Results:**

Among 162 patients (mean age 66 years, range 24–97; 57% male), 64% had a documented PC decision, with 43% of the total cohort having an early PC decision. Patients with an early PC decision had significantly fewer emergency department visits (10% vs. 25%; *p* = 0.015) and fewer hospitalizations (4% vs. 29%; *p* < 0.001) in their final month of life compared to those with a late/no decision. Overall, 34% of patients visited a dedicated PC unit, with a median of 93 days (range 5-619) from the first PC unit visit to death.

**Conclusions:**

An early PC decision significantly reduced acute hospital resource use at EOL among brain tumor patients. Nonetheless, approximately one-third of patients had no documented PC decision, and similarly low numbers had PC unit visits, highlighting ongoing gaps in timely PC initiation.

## Introduction

Primary brain tumors are often associated with a high symptom burden and a rapidly progressive course, leading to substantial morbidity and caregiver distress [1–3]. Although many patients receive multimodal treatments such as surgery, radiotherapy, and systemic therapies, the disease frequently recurs or becomes refractory, leaving patients with significant functional and cognitive impairments [1, 3, 4]. As the tumor advances, palliative care (PC) offers additional support by addressing symptom management, psychosocial needs, and care planning [5], yet it remains underutilized in neuro-oncology [6–10]. As a result, patients frequently require acute healthcare services, especially emergency department visits and hospitalizations, in their final weeks of life as symptoms rapidly worsen [11–13].

Early involvement of PC in other advanced cancers has been consistently linked to improved quality of life (QoL) and reduced aggressiveness of care at the end of life (EOL) [14–17]. However, robust evidence of such benefits in primary brain tumor populations is limited [1, 2, 18]. Across cancer populations more broadly, multiple structural and clinical barriers, including prognostic uncertainty, fragmented care pathways, and uneven access to PC services, have been shown to contribute to delayed referral and create disparities in timely PC integration [19, 20]. In glioblastoma, a SEER–Medicare analysis suggested that an earlier PC consultation was associated with outcomes such as fewer hospitalizations and increased hospice enrollment, reinforcing the potential value of timely PC in neuro-oncology [9]. Recent data from the EPCOG randomized clinical trial demonstrated that early integrated PC improved patient-reported outcomes in high-grade glioma but did not significantly reduce healthcare utilization [21].

In our single-center study of patients with malignant brain tumors in 2013–2014, we found that an early PC decision - terminating life‐prolonging anticancer treatment more than 30 days before death - was documented in 42% of cases and correlated with fewer emergency department visits and hospitalizations in the last month of life [22]. Given the ongoing need to minimize burdensome EOL care in this population, we conducted a new retrospective cohort study at our tertiary cancer center to explore how the timing of a PC decision relates to emergency department visits and hospital admissions among patients with primary brain tumors.

## Methods

### Cohort selection

This single-center, retrospective cohort study included adult patients (≥ 18 years) with a primary brain tumor treated at the Comprehensive Cancer Center of Helsinki University Hospital between January 2017 and December 2018. Patients who died between April 1, 2017, and December 31, 2018, were included, ensuring a minimum follow-up period of three months to comprehensively assess hospital resource utilization before death. Thus, patients who died before April 1, 2017 (*n* = 24), were excluded due to insufficient follow-up time. Additionally, three patients were excluded due to a concomitant second malignancy that was considered the probable cause of death. Patients were identified through hospital registries. The study was reported in accordance with the Strengthening the Reporting of Observational Studies in Epidemiology (STROBE) guidelines [23].

### Data collection

Data were extracted from electronic medical records, including demographics, date of death, date of last radiotherapy, hospital resource use (emergency department visits and hospitalizations) and PC unit visits. The timing of the PC decision was determined by searching for a Z51.5 diagnosis code or by a manual review of medical records.

### Palliative care terminology

In the Finnish healthcare system, an ICD-10 code Z51.5 is used when a PC decision has been made, indicating that cancer-directed life-prolonging treatments (e.g., chemotherapy) are discontinued and the treatment goal shifts to palliative intent. Because of this, Z51.5 reliably marks the timepoint of a documented PC decision. By contrast, PC outpatient clinic visits for symptom management or supportive care may occur while anticancer treatments continue, and these encounters are not coded with Z51.5. Patients were classified as having an early PC decision if it occurred > 30 days before death and late/no PC decision if it occurred ≤ 30 days before death or was not documented.

### Ethical statement

This retrospective study was done with the permission of the authorities of Helsinki University Hospital (HUS/325/2023). No patient interventions were performed. According to the Finnish legislation for research, no ethics committee approval was needed, as data used in the study consisted of deceased patients.

### Statistical analyses

All analyses were performed with IBM SPSS Statistics version 29 (IBM Corp., Armonk, NY, USA). Descriptive statistics are presented as median (range) for continuous variables and as numbers (percentages) for categorical variables. We compared early PC decision vs. late/no PC decision using Pearson’s chi-square or Fisher’s exact test for categorical variables. Continuous variables (e.g., time intervals) were compared with the Kruskal-Wallis test. Logistic regression analyses were performed to identify independent factors associated with emergency department visits and hospitalizations during the last 30 days of life. A p-value < 0.05 was considered statistically significant.

## Results

A total of 162 patients with primary brain tumor met the inclusion criteria. The mean age at death was 66 years (range 24–97), and 57% were male. Overall, 64% (103/162) had a documented PC decision. The characteristics of the patients are shown in Table 1.

**Table 1:** Patient characteristics according to timing of palliative care decision

Number of patients (%)	All patients (*n* = 162)	Early PC decision > 30 days before death (*n* = 70)	Late/no PC decision ≤ 30 days before death (*n* = 92)	*P*-value
Age at death median (range)	69 (24–97)	68 (31–97)	69 (24–93)	0.907*
Gender				0.704**
Male	93 (57)	39 (56)	54 (59)	
Female	69 (43)	31 (44)	38 (41)	
Palliative care decision	103 (64)	70 (100)	33 (36)	< 0.001**
Median time in days from palliative care decision to death (range)	44 (0-323)	62 (32–323)	13 (0–30)	< 0.001*

Abbreviations: n=number of patients, PC=palliative care. *Kruskal-Wallis Test. ** Pearson Chi-Square

### Palliative care decision

**Fig. 1:**
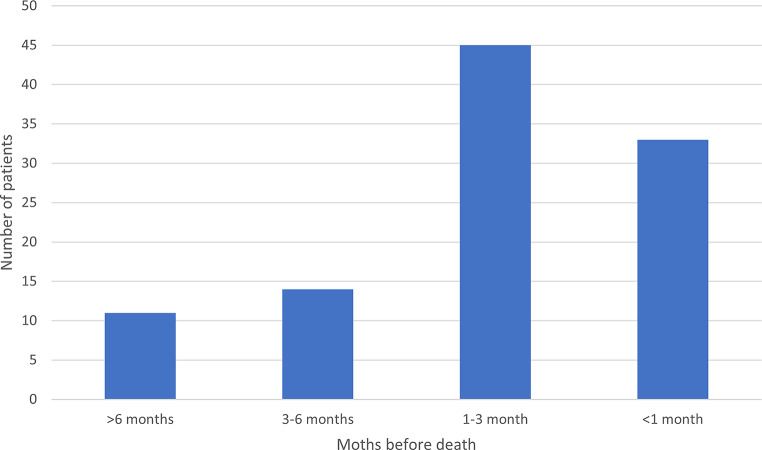
The timing of the palliative care decision before death

Among those with a PC decision, 70 patients (43% of the total cohort) had an early PC decision (> 30 days before death), while 33 (20%) had a late PC decision (≤ 30 days before death). The remaining 59 (36%) had no PC decision recorded. PC decision was made significantly earlier in patients with early PC decisions (median of 62 days, range 32–323 from decision to death) as compared to patients with late PC decisions (median of 13 days, range 0–30) (p < 0.001). The timing of the PC decision in relation to death is shown in Fig. 1.

### Use of acute hospital resources

In the last 30 days of life, 19% of the entire cohort visited the emergency department, and 19% were hospitalized. Patients with an early PC decision had significantly fewer emergency department visits (10% vs. 25%; p=0.015) and fewer hospitalizations (4% vs. 29%; p<0.001) than those with a late/no PC decision. For individuals requiring hospitalization, the median length of stay was seven days (range 1–23) for the total population. No significant difference was found between the study groups (Table 2).

**Table 2:** Use of hospital resources according to the timing of the palliative care decision

Number of patients (%)	All patients (*n* = 162)	Early PC decision > 30 days before death (*n* = 70)	Late/no PC decision ≤ 30 days before death (*n* = 92)	*P*-value
Emergency department visits within 60 days before death	70 (43)	26 (37)	44 (48)	0.174**
Emergency department visits within 30 days before death	30 (19)	7 (10)	23 (25)	0.015**
Hospitalizations within 60 days before death	61 (38)	23 (33)	38 (41)	0.272**
Hospitalizations within 30 days before death	30 (19)	3 (4)	27 (29)	< 0.001**
Inpatient days (median, range)	7 (1–23)	5 (2–23)	7 (1–21)	0.900*
Radiotherapy within 60 days before death	31 (19)	9 (13)	22 (24)	0.076
Radiotherapy within 30 days before death	11 (7)	< 5 (3)	9 (10)	0.083**
Median time in days from last radiotherapy to death (range)	134 (3-654)	231 (16–455)	100 (3-654)	< 0.001*

*Kruskal-Wallis Test. ** Pearson Chi-Square

The median time from last radiotherapy to death was significantly longer for patients with an early PC decision compared to those with a late/no PC decision (231 days vs. 100 days, p<0.001).

### Factors associated with emergency department visits and hospitalizations during the last 30 days of life

A late/no PC decision was significantly associated with higher odds of both emergency department visits and hospitalizations in the last 30 days of life. Compared with patients with an early PC decision, those with a late/no decision had almost a three-fold higher likelihood of emergency department visits (OR 2.99, 95% CI 1.19–7.51, p = 0.020) and nearly a nine-fold higher likelihood of hospitalization (OR 8.91, 95% CI 2.55–31.12, p < 0.001). Other covariates were not statistically significant. Regression results are presented in Table 3.

**Table 3:** Factors associated with emergency department visits and hospitalization during the last 30 days of life: logistic regression analysis

	OR	95% CI	*p*-value
A. Emergency department visits			
Age at death†	1.023	0.990–1.056	0.174
Gender (Ref. male)	0.830	0.358–1.923	0.663
Palliative care decision (Ref. early)	2.985	1.186–7.512	0.020
Palliative care unit visit	0.887	0.362–2.174	0.793
B. Hospitalizations			
Age at death†	1.021	0.987–1.057	0.220
Gender (Ref. male)	0.786	0.326–1.895	0.591
Palliative care decision (Ref. early)	8.914	2.554–31.119	< 0.001
Palliative care unit visit	0.470	0.168–1.315	0.150

Abbreviations: OR = odds ratio, CI = confidence interval, †Age at death was analyzed as a continuous variable

### Palliative care unit

Only 34% (n = 55) of patients visited a dedicated PC unit. The early PC decision group had a higher proportion of PC unit visits (41% vs. 28%; p = 0.080), with a significantly longer median interval between first PC unit visit and death (109 vs. 61 days; p = 0.015) compared to the late/no PC decision group (Table 4).

**Table 4:** Palliative care unit visits and timing measures

Number of patients (%)	All patients (*n* = 162)	Early PC decision >30 days before death (*n* = 70)	Late/no PC decision ≤ 30 days before death (*n* = 92)	*P*-value
Palliative care unit visit	55 (34)	29 (41)	26 (28)	0.080**
Median time (days) from a first palliative outpatient clinic visit to death (range)	93 (5-619)	109 (5-360)	61 (11–619)	0.015*

Abbreviations: n = number of patients, PC=palliative care

## Discussion 

In this retrospective single-center cohort study, we found that an early decision to terminate life-prolonging anticancer treatment, defined as a PC decision made more than 30 days before death, was significantly associated with fewer emergency department visits and hospitalizations at the EOL among patients with primary brain tumors. In multivariable logistic regression, the timing of the PC decision remained the strongest factor associated with acute care use. A late or no PC decision increased the odds of emergency department visits nearly three-fold and the odds of hospitalization almost nine-fold in the last month of life. Other covariates were not independently associated with acute care utilization. These findings reinforce existing evidence supporting timely integration of PC as an essential component of optimal neuro-oncology practice

Previous literature clearly indicates that patients with primary brain tumors frequently experience progressive neurological decline, necessitating increased acute healthcare services in their final weeks [3, 8, 10–13]. In our cohort, approximately 19% of patients visited the emergency department or were hospitalized in their last month of life, which aligns closely with international data indicating significant acute healthcare utilization in the final stages of brain tumor disease [10, 13, 24]. Neurological symptoms such as seizures and rapid clinical deterioration commonly drive these acute care visits [11, 22].

Our findings align with broader oncology literature demonstrating the benefits of early PC, particularly the reduction in acute hospital services at the EOL. A Cochrane systematic review by Haun et al. emphasized that early integration of PC for advanced cancer patients consistently reduces hospitalizations, aggressive care, and improves QoL [25]. Similarly, Diamond et al. reported that late referrals to PC services among brain tumor patients are associated with increased emergency visits and hospital resource utilization, highlighting the critical need for timely decision-making [26]. The recently published EPCOG trial by Golla et al. evaluated early integrated PC in patients with high-grade glioma but did not demonstrate significant differences in overall health care use between the intervention and control groups. This contrasts with our findings, where an early PC decision was associated with reduced acute care utilization at the end of life. The divergence likely reflects conceptual differences: in EPCOG, PC was integrated early while tumor-directed therapy continued, whereas a PC decision indicates a transition away from anticancer treatment to a palliative treatment intent. Consequently, ‘early integrated PC’ in EPCOG and ‘early PC decision’ in our study represent different stages of the illness trajectory, which may explain the differing impacts on health care use [21].

Despite these recognized benefits, our study revealed notable shortcomings in timely PC decision-making and referrals. Only 64% of our cohort had a documented PC decision, and just 43% were made sufficiently early (>30 days before death). No improvement is evident when compared to our earlier study from 2013–2014, where PC decisions were documented in 78% of cases, with early decision accounting for 42% of the entire population [22].

Further reflecting underutilization, only 34% of patients in our study visited a dedicated PC unit. This finding is consistent with international studies reporting low and delayed PC referrals among patients with brain tumors [6, 8, 10–12]. For example, Dullea et al. showed in a large US cohort of over 375,000 hospitalized patients with malignant brain tumors that only 15% received PC services [10]. Crooms et al. likewise showed that PC is often introduced only at advanced stages, despite the high symptom burden [6]. Nationally, brain tumor patients also remain underserved relative to other cancer populations. Haltia et al. (2023) reported that 37% of Finnish cancer patients overall received specialized PC, initiated a median of 112 days before death, while brain tumor patients had notably lower access rates [27]. In our cohort, the median interval from the first PC unit visit to death was 93 days, highlighting delayed referrals compared to other cancer groups. Recently published Finnish nationwide data confirm that brain tumor patients rarely access PC, although when they do, it significantly reduces hospitalizations at the EOL [24].

Direct comparisons of our findings with other international studies remain challenging, as few explicitly define or document a “PC decision.” However, evaluating anticancer treatments at the EOL provides a comparable perspective even though chemotherapy data were not studied in our cohort. In our cohort, 7% of patients received radiotherapy within 30 days before death, aligning with previous research indicating a continued tendency toward aggressive oncological interventions near death. A recent SEER-Medicare analysis showed substantial variability in the timing of treatment cessation among glioblastoma patients, with many continuing anticancer therapies close to death [9]. Similarly, international studies consistently report high rates of aggressive care near the EOL among brain tumor patients, including frequent emergency department visits, hospitalizations, and late discontinuation of active treatments [6, 12, 18]. Our findings reinforce these international observations and underline the persistent challenge of timely cessation of aggressive oncological treatments in neuro-oncology.

This study has several limitations. The dataset covers a relatively short and older time period (2017–2018), which may limit the timeliness and generalizability of the findings. As a single-center study, results may not fully reflect practices in other institutions or healthcare systems. The retrospective design also precludes causal inference, and some potentially relevant clinical information, such as disease severity, symptom burden, and patient preferences, was not available. Data on anticancer treatments at the EOL and on primary care service use were likewise lacking. Despite these limitations, the study includes all consecutive deceased adult brain tumor patients at a major oncology center, providing comprehensive real-world insights into PC decision-making and acute care use at the EOL. PC decision making was comprehensively evaluated by both searching electronic medical records and manual review.

## Conclusions 

Ultimately, little is known about the optimal model of PC in neuro-oncology. Our findings suggest that an early PC decision significantly reduces acute hospital resource utilization in patients with primary brain tumors. Moreover, an early PC decision appears to be interrelated with earlier access to specialized PC services, providing patients and families more time to benefit from supportive care interventions

## Data Availability

The data generated during the current study are not publicly available as the data is a part of the larger dataset owned by Helsinki University Hospital. Data are however available from the principal author Tiina Saarto upon reasonable request and with permission of Helsinki University Hospital.

## Abbreviations

EOL: End of life
PC: Palliative care
QoL: Quality of life
